# Assessing mpox epidemic readiness status in ECOWAS region: strengths, gaps, and recommendations for an improved response

**DOI:** 10.11604/pamj.supp.2025.50.1.46077

**Published:** 2025-03-02

**Authors:** Virgil Kuassi Lokossou, Andrew Sime Awori, Victor Fatimehin, Aishat Bukola Usman, Lionel Sogbossi, Felix Agbla, Melchior Athanase Aïssi, Issiaka Sombie

**Affiliations:** 1 West African Health Organization, Bobo-Dioulasso, Burkina Faso, Nigeria; 2 ECOWAS Regional Center for Surveillance and Disease Control, Abuja, Nigeria

**Keywords:** Mpox epidemic, readiness, preparedness and response, ECOWAS

## Abstract

Mpox (formerly known as monkeypox) has re-emerged as a significant public health threat in West Africa, creating new challenges for regional and national health systems. Drawing from past outbreaks, the West African Health Organization (WAHO) has been proactive in enhancing public health preparedness, aligned with WHO recommendations. This study aims to assess the readiness of the ECOWAS region to manage the ongoing mpox outbreak, focusing on critical areas of national epidemic preparedness and response. A survey using the WHO mpox-epidemic readiness checklist tool was conducted in 14 ECOWAS Member States from August 25 to September 15, 2024. Key stakeholders, including Directors of Public Health and Heads of National Public Health Institutes, participated in the assessment. Mpox preparedness meetings and training reports from WAHO were also reviewed to evaluate the region’s efforts. A thematic analysis identified key themes, strengths, challenges, and gaps in mpox readiness. The findings revealed considerable variations in readiness across ECOWAS countries. Strengths include established coordination structures, operational surveillance systems, and developed risk communication plans. However, there are major gaps in laboratory capacity, case management, and logistics. Countries such as Liberia and Guinea-Bissau lack sufficient healthcare workers and ICU facilities for managing severe mpox cases. In Senegal and The Gambia, inadequate adaptation and translation of Information, Education, and Communication (IEC) materials hamper public awareness. Logistic challenges, particularly the absence of emergency supply mechanisms and cold chain capacity, also hinder effective responses. Despite progress in mpox preparedness, ECOWAS countries must address significant gaps in laboratory capacity, healthcare infrastructure, and logistics through enhanced coordination and investments.

## Introduction

The mpox virus, formerly known as monkeypox, was first identified in 1958 during outbreaks of a pox-like illness in colonies of monkeys kept for research purposes, which led to its initial naming [1]. Human cases of mpox were first reported in 1970, in the Democratic Republic of the Congo (DRC), marking the beginning of its recognition as a zoonotic disease of significant public health concern in Africa [2]. Since then, sporadic outbreaks have occurred predominantly in Central and West Africa, with the virus becoming endemic in several countries across these regions. The causative agent of mpox belongs to the Orthopoxvirus genus, sharing close genetic relations with smallpox and cowpox viruses [3].

Historically, mpox was largely restricted to rural, forested areas in Africa, where human transmission occurred through close contact with infected wildlife, particularly rodents [4]. Limited access to healthcare and infrastructure, combined with poor surveillance systems, allowed the disease to persist in these remote communities for decades [5]. However, a significant shift occurred in 2022 when mpox spread globally, marking a dramatic change in its epidemiology. This spread occurred mainly through human-to-human transmission, primarily in sexual and social networks that were previously unexposed [6,7]. By July 2022, the World Health Organization (WHO) declared mpox a Public Health Emergency of International Concern (PHEIC), signaling its emergence as a global health threat for the first time.

Three distinct waves can characterize the epidemiological history of mpox. The first wave, spanning from the 1970s to the 2010s, saw localized outbreaks in rural African regions. These were driven by zoonotic transmission with minimal human-to-human spread, and the outbreaks remained largely undetected due to inadequate surveillance and health infrastructure [8,9]. A second wave emerged in the 2010s, particularly affecting Nigeria, where rising urbanization, environmental encroachment, and cross-border trade created favourable conditions for increased human transmission. The third and most recent wave began in 2022, when mpox spread beyond endemic regions, affecting over 100 countries globally and necessitating urgent global coordination to manage the outbreak.

As of 2024, West Africa continues to experience mpox outbreaks, particularly in Nigeria, Liberia, and Ghana, where community transmission remains a concern. By mid-August 2024, both the WHO and the Africa Centres for Disease Control and Prevention (Africa CDC) had declared the resurgence of mpox a significant public health emergency. The WHO reclassified mpox as a PHEIC on August 14, 2024, while the Africa CDC designated it a Public Health Emergency of Continental Security (PHECS) on August 13, 2024. As of September 16, 2024, the Economic Community of West African States (ECOWAS) region reported 131 confirmed cases, including one death, across four active member states. Nigeria accounted for 67 cases across 23 states and the Federal Capital Territory; Côte d’Ivoire reported 52 cases, including one death; Liberia recorded 11 confirmed cases from five counties; and Guinea recently confirmed its first case.

In comparison to non-endemic regions where mpox vaccination and treatment strategies have been more accessible, African countries face persistent challenges. These include limited vaccine access, constrained health infrastructure, underdeveloped surveillance and laboratory systems, and low levels of public awareness and community engagement. The threat of zoonotic spillover continues to be exacerbated by rapid urbanization and environmental degradation in West Africa, sustaining mpox as an ongoing risk in the region [10,11]. The current landscape underscores the need for strengthened, coordinated efforts in readiness and response [12]. The West African Health Organization (WAHO) has been instrumental in these efforts, spearheading initiatives to bolster surveillance, enhance case management, and support the development and implementation of national mpox preparedness plans. WAHO, in collaboration with partners like the World Bank´s Health Security and Resilience Project and the United States Agency for International Development (USAID), has reinforced regional coordination through capacity-building workshops, inter-governmental meetings, and simulation exercises to prepare health systems for potential outbreaks.

This paper aims to analyze mpox epidemic readiness efforts within ECOWAS member states and provide recommendations for guiding preparedness and response strategies in collaboration with both regional and global partners for future public health threats.

## Methods

**Study design:** this was a rapid assessment conducted between August 25 and September 15, 2024, to assess the readiness of the 15 ECOWAS Member States for mpox epidemic preparedness and response. The study utilized the WHO mpox Epidemic Readiness Checklist as the primary data collection tool to evaluate key preparedness pillars across the region.

### Data collection

**Desk review:** a desk review was conducted to analyze national situation reports, epidemic preparedness and response plans, and outbreak response documentation. Reports submitted to WAHO, along with minutes from mpox preparedness activities, were also reviewed to provide context for readiness assessments.

**WHO readiness checklist:** the WHO mpox Epidemic Readiness Checklist was sent to key stakeholders in each ECOWAS Member State. This tool evaluates preparedness across several pillars, including: i) incident command and coordination; ii) risk communication and community engagement; iii) points of entry (PoEs); iv) case management; v) infection prevention and control (IPC); vi) logistics and supply management. Stakeholders selected to complete the checklist included directors of public health, senior technical advisors, laboratory directors, and managers of epidemic preparedness and response teams. Individuals also involved in infection prevention and control, community engagement, and logistics were specifically targeted due to their critical roles in the mpox response.

**Data analysis:** responses from the readiness checklists and findings from the desk review were synthesized and analyzed using thematic analysis. This approach identified key themes, strengths, gaps, and challenges in the region’s mpox preparedness. Data were presented in aggregate to maintain confidentiality, with illustrative examples drawn from individual countries where permission was granted. Visual tools such as heat maps, tables, and graphs were used to display readiness levels and highlight variations across countries.

**Ethical considerations:** Informed consent was obtained from participating countries through their National Public Health Institutes. The purpose and scope of the readiness assessment were explained, and participation was voluntary. Individual stakeholders also provided informed consent, with assurances of confidentiality and the right to withdraw at any point. Data were anonymized during analysis to ensure privacy. Specific quotes or country-level examples were included in the findings only with explicit permission from respondents.

## Results

### Coordination

Effective coordination is fundamental to the success of any public health response, including the ongoing mpox outbreak in the ECOWAS region. The coordination pillar focuses on how well-prepared countries are in terms of their national plans, multi-sectoral partnerships, emergency operations centers, financial resources, and technical advisory groups. This pillar ensures that all relevant sectors and stakeholders such as health, agriculture, environment, and finance work together seamlessly to implement strategies, allocate resources, and provide the leadership needed for a timely and effective response. Across the ECOWAS region, coordination mechanisms were in place to varying degrees, but gaps remain in key areas. Thirteen (92%) countries have functional PHEOCs that can be activated promptly, and multi-sectoral coordination mechanisms exist across ministries in all member states. However, four countries, including Guinea-Bissau and Cabo Verde, reported challenges in accessing emergency funds and Cabo Verde lacks a national preparedness plan, reducing its capacity to address outbreaks effectively (Table 1).

**Table 1:** assessment of coordination readiness for mpox response in the ECOWAS region

Country	National preparedness and response plans	Multi-sectoral coordination	Public Health Emergency Operations Centre (PHEOC)	Funding for emergency preparedness and response	National Immunization Technical Advisory Group (NITAG)
Benin	Yes	Yes	Yes	Yes	Yes
Burkina Faso	Yes	Yes	Yes	Yes	Yes
Cabo Verde	No	Yes	No	No	No
Côte d'Ivoire	Yes	Yes	Yes	No	Yes
Gambia	Yes	Yes	Yes	No	Yes
Ghana	Yes	Yes	Yes	No	Yes
Guinea	Yes	Yes	Yes	No	Yes
Guinea Bissau	Yes	Yes	Yes	No	Yes
Liberia	Yes	Yes	Yes	No	Yes
Mali	Yes	Yes	Yes	Yes	Yes
Niger	**Information not available**				
Nigeria	Yes	Yes	Yes	Yes	Yes
Senegal	Yes	Yes	Yes	Yes	Yes
Sierra Leone	Yes	Yes	Yes	No	Yes
Togo	No	Yes	Yes	No	Yes

### Surveillance

**Figure 1:**
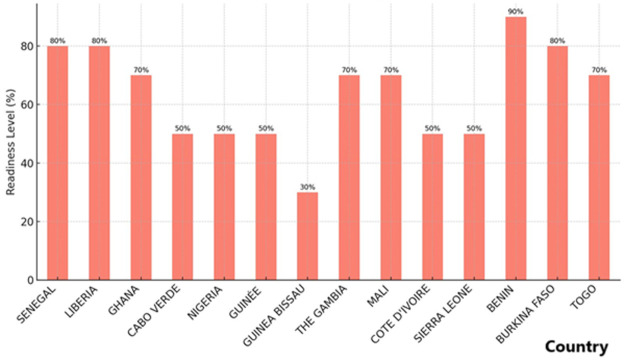
surveillance readiness level for mpox among ECOWAS member states

Surveillance systems are vital for early detection, prompt response, and effective management of public health threats like mpox. In the ECOWAS region, surveillance activities encompass event-based monitoring, the use of standardized case definitions, communication with healthcare professionals, and integration with WHO tools. Proper training of health workers and collaboration with both public and private sectors are also key components of a comprehensive surveillance strategy. While several countries have established functional surveillance mechanisms, significant gaps in areas like training and the inclusion of private healthcare providers remain. Addressing these weaknesses is essential for improving the overall effectiveness of mpox surveillance. i) All 14 responding countries have adopted the WHO mpox case definition, ensuring consistent disease reporting. Eleven (91.7%) of the countries have integrated WHO surveillance tools, enhancing their data collection and reporting frameworks. ii) Only 50% of the countries have trained health workers in mpox surveillance, leaving critical gaps in Liberia, Guinea-Bissau, and Côte d’Ivoire. iii) Event-based monitoring (EBM) systems were absent in Cabo Verde and Côte d´Ivoire, reducing real-time outbreak detection capacities (Figure 1).

### Rapid response capacities

The rapid response teams (RRTs) are crucial for promptly addressing public health emergencies like the mpox outbreak. The readiness of RRTs, both at the national and sub-national levels, as well as their integration with Emergency Operations Centres (EOCs) and Incident Management Systems (IMS), determine the overall effectiveness of the public health response. While ECOWAS member states have made strides in establishing RRTs, certain gaps in communication and coordination remain, particularly at the local levels. Addressing these shortcomings will enhance the region’s ability to respond quickly and effectively to mpox and future outbreaks.

*Operational status of RRTs:* all 15 ECOWAS Member States have fully operational RRTs at the national level, demonstrating a comprehensive commitment to mpox preparedness and response.

*Sub-national level:* several countries lack RRTs at the sub-national level, which limits the efficiency of localized responses. This gap may cause delays in outbreak management and reduce the overall capacity to handle mpox cases effectively.

**Figure 2:**
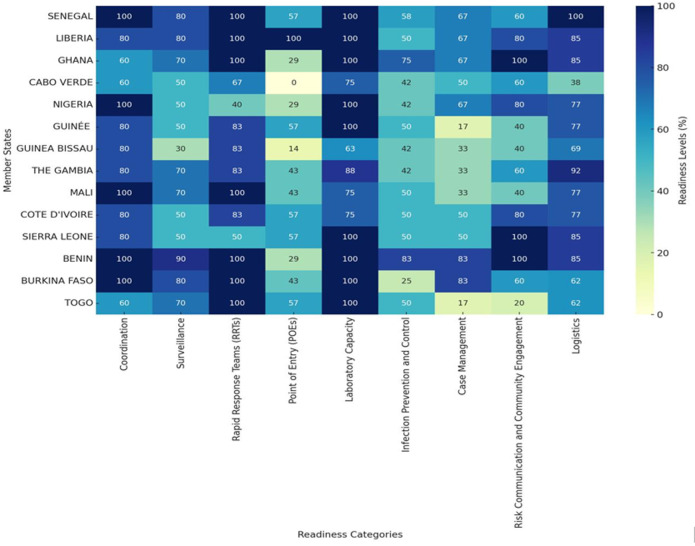
heatmap comparing the readiness levels across ECOWAS member states and various indicators; the colors represent the level of preparedness, graded in percentages, with darker shades indicating higher readiness at 100% and lighter shades indicating lower readiness down to 0%

*Communication and coordination gaps:* critical communication gaps have been identified between RRTs, Emergency Operations Centers (EOCs) and Incident Management Systems (IMS), particularly in The Gambia. These gaps could hinder effective coordination during emergencies, affecting timely and efficient responses to mpox outbreaks (Figure 2).

### Laboratory capacity

Laboratory capacity is a critical component in the detection and management of infectious diseases like mpox. Across ECOWAS, the ability to accurately and safely diagnose mpox through PCR platforms, manage laboratory biosafety, and ensure secure sample transport plays a pivotal role in containment and response efforts. While most member states have established solid foundations for viral detection and sample handling, gaps in equipment and resources in a few countries pose risks that could affect overall preparedness.

*PCR platform for detection of viral pathogens:* all ECOWAS member states have operational PCR platforms for detecting viral pathogens, including mpox, ensuring accurate and timely diagnosis across the region.

*Laboratory equipment for biosafety and waste management:* most countries have essential biosafety equipment, but Guinea-Bissau lacks an autoclave, increasing the risk of biohazardous waste mismanagement.

*Sample transport for infectious materials:* all countries have trained personnel for transporting infectious samples, but Guinea-Bissau, Cabo Verde, Gambia, and Côte d’Ivoire face challenges in acquiring necessary packaging materials.

*Standard operating procedures (SOPs):* most countries have SOPs for sample collection and transport, but Cabo Verde lacks these critical guidelines, potentially compromising sample integrity.

### Infection prevention and control (IPC)

Infection prevention and control (IPC) is crucial for managing the spread of mpox within healthcare facilities. Effective IPC programs help minimize nosocomial infections, safeguard healthcare workers, and ensure proper triage and isolation of suspected cases. The readiness of ECOWAS countries in this pillar varies, with key gaps in several areas, such as IPC programs, PPE availability, and training of healthcare workers.

*Functional IPC programmes:* operational national IPC programs were in place across most ECOWAS countries; however, Cabo Verde, Burkina Faso, and Guinea-Bissau lack such programs, which limits coordinated infection control efforts.

*Triage systems:* effective triage systems were missing in Senegal, Cabo Verde, Guinea-Bissau, Liberia, and The Gambia, impairing the timely identification and isolation of suspected mpox cases.

*Standard precautions:* the implementation of standard precautions, such as hand hygiene and personal protective equipment (PPE) use, is inconsistent across ECOWAS nations, increasing the risk of mpox transmission.

*Isolation facilities:* none of the member states have designated isolation units or beds specifically for mpox patients, leaving gaps in preventing healthcare-associated transmission.

*Personal protective equipment availability:* critical shortages of PPE were reported in Liberia, Ghana, Nigeria, Guinea-Bissau, Gambia, Sierra Leone, and Côte d’Ivoire, compromising the safety of healthcare workers managing mpox cases.

*Infection prevention and control training:* most countries provide IPC training; however, Guinea-Bissau lacks this critical training for healthcare workers, exposing the system to vulnerabilities in managing mpox cases.

*Monitoring protocols:* Liberia, Cabo Verde, Guinea-Bissau, Togo, and Mali do not have dedicated IPC teams to monitor healthcare workers exposed to mpox cases, increasing the risk of secondary transmission.

**Figure 3:**
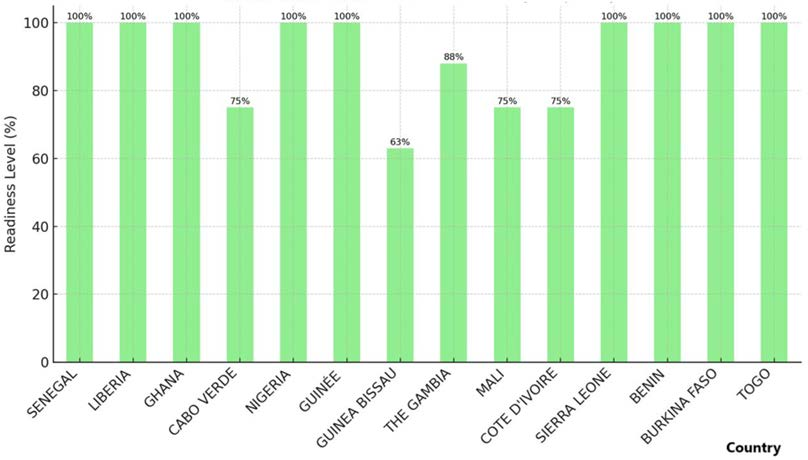
infection prevention and control readiness level for mpox among ECOWAS member states

*Exposure management policies:* several countries, including Guinea-Bissau, Senegal, Guinea, Sierra Leone, and Côte d’Ivoire, lack policies for managing healthcare workers exposed to confirmed mpox cases, heightening the risk of inadvertent transmission within healthcare settings (Figure 3).

### Case management

Effective case management is critical for reducing the severity of mpox cases, minimizing transmission risks, and preventing complications. Adequate training, transport systems, and ICU capacity are essential for a well-rounded healthcare response to mpox outbreaks. The readiness of ECOWAS countries in this area shows gaps, particularly in training and healthcare infrastructure, which could hinder effective case management.

*mpox case management training:* Senegal, Nigeria, and Benin have initiated training (60% readiness). Liberia, Guinea-Bissau, Gambia, Guinea, Sierra Leone, and Côte d’Ivoire still lack this essential training.

*Ambulance services:* Guinea-Bissau and Togo have dedicated ambulance services (13% readiness), but most other countries do not, increasing the risk of transmission.

*Referral health facilities:* Senegal, Ghana, and Nigeria have designated mpox referral centers (80% readiness), while Guinea-Bissau, Togo, and Mali lack these facilities.

Intensive care unit (ICU) capacity: Côte d’Ivoire, Ghana, and Nigeria have adequate ICU facilities (40% readiness). Other countries, including Liberia, Cabo Verde, and Guinea, have insufficient ICU capacity.

### Point of entry (PoE) readiness

Point of entry readiness is critical in preventing the cross-border spread of infectious diseases like mpox. Effective management at borders ensures that suspected cases are promptly detected, isolated, and managed to prevent outbreaks. Key components of PoE readiness include having public health emergency plans, trained personnel, screening and isolation facilities, safe transport mechanisms, and clear communication strategies.

*Public health emergency plan at PoEs:* only Ghana, Nigeria, Liberia, Togo, and Côte d’Ivoire have public health emergency plans for major PoEs, representing 33.3% readiness. Other countries without these plans risk delayed responses during outbreaks.

*Training of staff on managing sick passengers:* seventy-five (75%) of countries have provided sufficient training for PoE staff, although Ghana, Cabo Verde, and Gambia have not yet done so, and Nigeria lacks supervision for its personnel.

*Training of staff on managing sick passengers:* seventy-five (75%) of countries have provided sufficient training for PoE staff, although Ghana, Cabo Verde, and Gambia have not yet done so, and Nigeria lacks supervision for its personnel.

*Mpox screening and isolation facilities at PoEs:* screening and isolation facilities are in place in several countries, including Senegal, Liberia, Guinea, Guinea-Bissau, and Gambia, helping contain potential outbreaks. Broader implementation of screening across the region is still required.

*Mechanism for transporting suspected cases:* eighty percent (80%) readiness is noted for safe transport protocols, but gaps exist in Cabo Verde, Nigeria, and Guinea-Bissau, where there are no mechanisms for transferring suspected cases from PoEs to health centers.

**Figure 4:**
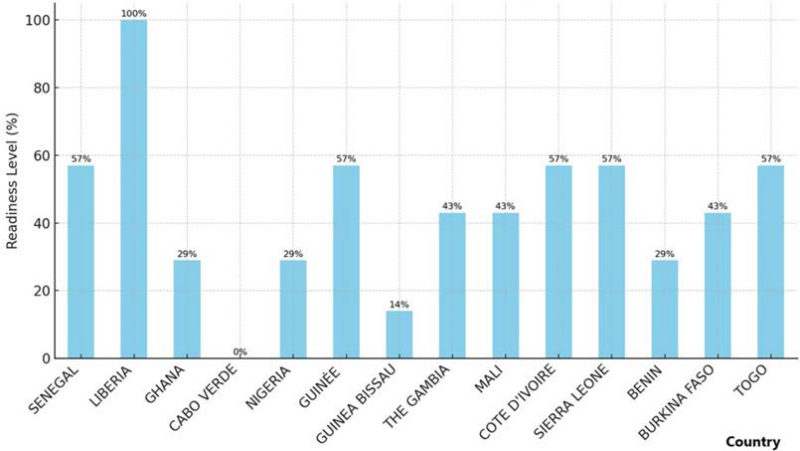
points of entry readiness level for mpox among ECOWAS member states

*Training in mpox risk communication:* Liberia is the only country with trained PoE staff in mpox risk communication (8.3% readiness), highlighting a critical gap in most countries, where staff is not adequately prepared to provide key information to travelers (Figure 4).

### Risk communication and community engagement

Effective risk communication is crucial in public health emergencies like mpox, as it ensures timely information dissemination to the public, builds trust, prevents misinformation, and engages communities in adopting preventive measures. This pillar assesses the ability of countries to manage communication during outbreaks, focusing on the availability of strategic plans, community involvement, and management of misinformation.

*Functioning risk communication structures:* all ECOWAS member states have established risk communication structures and strategic plans, reflecting their preparedness to inform the public during health emergencies.

*Adaptation and translation of IEC materials:* Ghana, Nigeria, and Côte d’Ivoire have adapted and translated information, education, and communication (IEC) materials to local languages (60% readiness). However, countries such as Senegal, Guinea, Guinea-Bissau, Gambia, Togo, and Mali have yet to complete these adaptations, limiting effective outreach to diverse populations.

*Distribution of IEC materials:* countries like Ghana, Sierra Leone, and Benin have successfully distributed IEC materials at critical locations, including health facilities and points of entry. This helps in raising awareness and ensuring that people recognize symptoms and know preventive measures.

*Misinformation management:* Cabo Verde and Togo lack systems for managing misinformation, such as monitoring tools or hotlines. These mechanisms are vital for addressing public concerns and preventing rumors from spreading during outbreaks.

*Regular public updates:* countries such as Guinea, Guinea-Bissau, Burkina Faso, Togo, and Mali do not have plans for consistent public updates on mpox. Regular updates from trusted sources are necessary to keep communities informed and engaged in outbreak prevention.

### Logistics

Logistics plays a critical role in the mpox response, ensuring the timely procurement, storage, and distribution of essential supplies such as PPE, diagnostics, vaccines, and other medical necessities. This pillar assesses the readiness of countries to handle logistical challenges in crises, highlighting both strengths and gaps.

*Emergency supply mechanisms:* some countries, including Mali, have designated logistics and procurement focal points, improving coordination. However, Cabo Verde and Guinea-Bissau lack emergency supply mechanisms, delaying critical supplies during outbreaks.

*Storage capacity:* Guinea has some cold chain capacity, but Cabo Verde and Guinea face significant storage limitations, especially for vaccines, affecting the quality of response efforts.

*Distribution of PPE and IPC materials:* although some countries have distributed PPE, Liberia, Guinea-Bissau, Mali, and Côte d’Ivoire lack pre-positioned stocks, delaying rapid response. Cabo Verde and Côte d’Ivoire also lack clear distribution plans.

*Transport mechanisms:* while some countries have functioning transport systems, Cabo Verde and Guinea-Bissau face underdeveloped logistics and Nigeria and Benin report delays due to visa-on-arrival issues for emergency teams.

*Stock management systems:* Guinea and Guinea-Bissau lack stock management systems, hindering real-time tracking and effective distribution of supplies during mpox outbreaks (Table 2, Table 2.1).

**Table 2:** highlighting strengths, gaps, and recommendations on thematic areas

Thematic areas	Strengths	Gaps	Recommendations
**Coordination**	National preparedness: 92% (12/15) of member States have national mpox preparedness and response plans, benefiting from prior health crisis experiences; all 14 countries maintain functional multi-sectoral coordination mechanisms across ministries and sectors; PHEOCs: 92% of countries can activate Public Health Emergency Operations Centres (PHEOCs) within 120 minutes.	Lack of preparedness plans, funding barriers, and delays in accessing emergency funds.	Develop national preparedness and response plans for mpox; strengthen financial readiness by improving emergency fund allocation mechanisms.
**Surveillance**	WHO case definitions: all 14 member states use the WHO surveillance case definition for mpox, ensuring consistency in detection and reporting; WHO surveillance tools: most countries, except Cabo Verde, have adopted WHO surveillance tools, facilitating standardized reporting and regional coordination; event-Based Monitoring (EBM): 83% of member states have EBM systems, enabling real-time outbreak detection and analysis.	The absence of EBM systems in some countries limits their ability to promptly detect mpox outbreaks; lack of communication of case definitions to health professionals, risking delays in case identification and reporting; absence of mpox surveillance training for health workers; private sector integration: nearly 30% of countries have not incorporated the private sector into surveillance systems, which may lead to underreporting of mpox cases.	Implement EBM systems in member states; ensure that updated mpox case definitions are rapidly disseminated to healthcare providers in both the public and private sectors; accelerate training of health workers in mpox surveillance in countries that are lagging to improve early detection and response efforts; integrate the private healthcare sector into national surveillance systems to ensure comprehensive data collection and rapid case identification.
**Rapid response capacity**	National level RRTs: all 15 ECOWAS states have operational RRTs, showing strong mpox preparedness with diverse expertise for complex outbreaks; team composition: RRTs comprise clinicians, infectious disease specialists, biologists, and risk communication experts, ensuring comprehensive response capabilities.	Lack of regional RRTs; event-based information and response (EIR). Mechanism inactivation; linkage between RRTs and EOCs/IMS; insufficient PPE.	Operationalize RRTs at regional and provincial levels to strengthen local response capacity; prioritize activating the EIR mechanism in Guinea for faster mpox outbreak detection and response; strengthen linkages between RRTs and EOCs/IMS; provide Adequate PPE.
**Point of entry (POE)**	Established screening and isolation facilities; proactive PoE screening.	Lack of comprehensive public health emergency plans at PoEs; insufficient staff training on managing sick passengers; lack of established transport mechanisms for suspected cases; limited training in risk communication.	Develop and implement public health emergency plans at PoEs; enhance training for PoE staff; establish Safe transport mechanisms for suspected cases; expand risk communication training:
**Laboratory capacities**	PCR platform availability; availability of PPE for laboratory personnel; training in infectious material transport: personnel in all member states are trained in safely transporting infectious materials, ensuring secure sample transfer between laboratories and across borders for mpox response.	The lack of biosafety cabinets presents a biosafety risk; insufficient waste infrastructure risks environmental contamination and mpox transmission; absence of triple packaging, and missing standard operating procedures (SOPs) in Cabo Verde.	Provision of biosafety components: enhancing waste management in member states; Countries lacking triple packaging should be supplied with these materials to ensure the safe transport of infectious samples to reference laboratories for confirmatory testing; development of SOPs in Cabo Verde.

**Table 2.1:** highlighting strengths, gaps, and recommendations on thematic areas

Infection prevention and control (IPC)	National IPC programmes: several countries have functional national IPC programs that help coordinate infection control measures; standard precautions: most member states apply hand hygiene, PPE, and waste management protocols, the key to preventing mpox transmission; IPC training: many countries have trained healthcare workers in IPC measures, improving readiness to manage mpox cases.	No national IPC programmes in some countries in the region; Absence of mpox-specific guidelines; non-functional triage; no isolation units: all countries lack dedicated isolation units or beds for mpox patients; some member states lack systems for safe medical waste disposal; several countries lack systems to monitor healthcare workers exposed to mpox.	Establish IPC programmes; develop mpox-Specific guidelines: countries should create and implement comprehensive IPC guidelines for mpox; improve PPE Access; enhance triage and isolation: ensure functional triage systems and establish isolation units to manage mpox cases.
**Case management**	Dedicated ambulance services: some countries have dedicated ambulance services for mpox, which is essential for the safe transport of suspected cases; ambulance crew training: only one country has trained ambulance crews specifically for handling mpox patients, ensuring safe transport and minimizing transmission risks.	Healthcare workers in some member states lack training in mpox case management; limited ICU capacity; absence of referral health facilities; shortage of infectious disease specialists; inadequate ambulance services and crew training.	Prioritize training healthcare workers in case management for mpox, particularly in countries with significant gaps; expand ICU and critical care facilities; establish referral centers; recruit infectious disease specialists; train ambulance crews.
**Risk communication and community involvement**	Established risk communication structures: all ECOWAS member states have functioning risk communication structures and strategic plans, ensuring timely and accurate dissemination of information; effective IEC distribution: some countries have successfully distributed Information, Education, and Communication (IEC) materials at strategic points, enhancing public awareness.	Adaptation and translation of IEC materials to reach diverse populations effectively; the mechanism for managing rumours; public engagement plans.	Prioritize the adaptation and translation of mpox-related communication materials into local languages to ensure wider public reach and understanding; establish misinformation management mechanisms; and enhance public engagement.
**Logistics**	Functioning supply chain in some countries: some countries have functional logistics frameworks, enabling timely interventions during outbreaks; established logistics structures: certain ECOWAS countries have designated logistics and procurement focal points, ensuring better coordination with international supply chains.	Absence of designated logistics and procurement focal points; some countries lack mechanisms for mobilizing emergency supplies, leaving them vulnerable during crises; storage and cold chain management: a few countries in the region face insufficient storage capacity for essential items like vaccines, compromising their ability to respond effectively; countries that lack pre-positioned PPE and IPC materials, limit rapid response capabilities; poor transport mechanisms; lack of isolation unit capacity in some countries.	Countries without logistics and procurement focal points should appoint them immediately; develop emergency supply mechanisms; enhance storage capacity: particularly cold chain management, to maintain the efficacy of temperature-sensitive supplies like vaccines; pre-position critical stocks: countries without pre-positioned PPE and IPC materials should build up these reserves to ensure they can respond rapidly to outbreaks; improve transport mechanisms; strengthen isolation units: invest in both infrastructure and staffing to ensure the availability of functional isolation units in Cabo Verde and Cote d'Ivoire.

## Discussion

The response to the ongoing mpox outbreak in the ECOWAS region reflects a blend of preparedness and significant challenges. Across the 15 member states, most have instituted strong foundational structures for public health emergencies, as seen in their established Public Health Emergency Operations Centres (PHEOCs) and surveillance mechanisms. However, critical gaps in logistics, rapid response capabilities, laboratory capacity, and infection prevention and control (IPC) measures pose considerable risks to the region´s overall ability to manage the epidemic effectively [5,13]. A related report from the WHO and public health bodies, including Africa CDC, observed similar weaknesses in surveillance, laboratory capacities, and infection prevention practices that are common across the region. It emphasizes the need to strengthen these areas to improve outbreak response capabilities [14].

The surveillance systems in place for mpox within the ECOWAS region highlight both commendable efforts and considerable shortcomings. Countries such as Nigeria and Ghana have shown robust surveillance frameworks, integrating public health data from both governmental and non-governmental actors. However, key weaknesses, such as the inconsistent training of healthcare workers in case definition and the limited involvement of the private sector, undermine these efforts. These issues are particularly pressing given the cross-border nature of outbreaks. For example, Guinea and Côte d’Ivoire have shown limited surveillance coordination with neighboring countries, which hinders effective regional collaboration.

To strengthen surveillance, countries must address training gaps and improve information dissemination on case definitions and reporting systems. Research shows that one of the critical facilitators for effective disease surveillance in Africa is addressing training and operational gaps in public health workforce skills, particularly in using digital surveillance tools [14]. Strengthening information dissemination through digital platforms ensures real-time data flow, improving the detection and tracking of disease outbreaks. Moreover, digital surveillance tools demonstrated effectiveness in providing more accurate and timely data, essential for early detection and containment of outbreaks [15]. The inclusion of the private sector, often the first point of contact for many patients, will also enhance real-time surveillance capabilities, as private health facilities account for a significant portion of initial consultations in many African countries [16]. Expanding the use of these digital systems across public and private healthcare sectors will improve reporting accuracy and speed in the region´s healthcare systems [17].

Rapid response teams (RRTs) have been established in all ECOWAS countries, with strong coverage in Ghana, Senegal, and Benin. These countries have demonstrated the ability to quickly mobilize teams to conduct field investigations and deploy infection control measures. However, gaps in operational readiness-particularly in the provision of personal protective equipment (PPE) and emergency infection response (EIR) activation-have been observed across many countries, including Mali and Guinea-Bissau. In Liberia and Guinea, RRTs are hindered by limited resources, such as the availability of transport and pre-positioned medical supplies. These challenges were also highlighted in the response to outbreaks in Guinea and Liberia, where logistical issues delayed the rapid response team (RRT) mobilization [18]. Enhancing RRTs requires investment in operational readiness. Regular simulation exercises, along with pre-positioning of PPE and other essential equipment, will improve the speed and effectiveness of RRT deployment during outbreaks. Regional coordination must also be strengthened to enable countries to share resources, expertise, and personnel during emergencies.

Laboratory capacity for mpox diagnosis varies significantly across the region. While many countries have PCR platforms for viral detection, infrastructure gaps, particularly regarding biosafety and cold chain storage, remain critical concerns. Côte d’Ivoire´s lack of a biosafety cabinet, Guinea-Bissau´s absence of autoclaves, and widespread shortages of triple packaging materials for safe sample transport severely limit the region´s ability to conduct safe and effective diagnostics. Moreover, reliance on centralized PCR testing labs delays results turnaround times, especially in countries with poor transport infrastructure like Guinea and Mali. As mpox diagnosis depends on timely PCR testing, decentralizing diagnostic capacity using portable PCR platforms like Cepheid Xpert could significantly enhance the speed of case identification [19,20]. Promoting point-of-care (POC) diagnostics, such as lateral-flow and loop-mediated isothermal amplification (LAMP) tests, could enable faster diagnosis, particularly in remote or resource-limited settings [21]. Collaborating with international partners to support the deployment of these technologies, as well as artificial intelligence applications for mpox lesion diagnosis, would enhance laboratory readiness.

Infection prevention and control (IPC) efforts are foundational to reducing mpox transmission. However, inconsistencies in healthcare worker training, IPC materials, and functional isolation units persist across ECOWAS member states. For instance, Cabo Verde lacks isolation unit standards, while Côte d’Ivoire struggles with staffing and resource allocation for case management. Addressing these deficiencies is critical for effective IPC. Ensuring adequate training on mpox-specific IPC measures, alongside investments in PPE and other IPC materials, is essential to protect healthcare workers and the general population. Studies show that appropriate PPE use, including gloves, masks, and gowns, significantly reduces the risk of transmission in healthcare settings [22]. Strengthening ICU capacities and isolation units across the region, particularly in under-resourced countries like Guinea-Bissau and Sierra Leone, will bolster the region´s ability to contain mpox outbreaks within healthcare settings [23]. Effective contact tracing and isolation practices are also key to preventing the spread of the virus [24].

Effective communication is paramount in any public health emergency, and mpox is no exception. While most ECOWAS countries have established risk communication structures, the translation and adaptation of information, education, and communication (IEC) materials into local languages remain a major challenge. Countries like Senegal, Gambia, Guinea, and Mali have not adequately translated these materials, which limits their ability to engage linguistically diverse populations. Additionally, the absence of rumour management mechanisms in countries like Cabo Verde and Togo presents a significant vulnerability. In the age of social media, misinformation spreads rapidly, often leading to public confusion, non-compliance, and stigma against affected individuals. Establishing rumour monitoring tools and public hotlines will be critical for managing misinformation and maintaining public trust during the mpox outbreak. Community engagement strategies must also be scaled up to involve local leaders, community health workers, and civil society in disseminating accurate information and promoting adherence to health measures. Engaging communities in decision-making around vaccination and risk communication and community engagement (RCCE) strategies foster trust and cooperation. It is also essential to clarify vaccination strategies and engage diverse community groups to prevent misinformation and rumours, which have previously spread due to poor communication [25]. Empowering communities to take part in outbreak control efforts through regular public updates, engagement sessions, and accessible IEC materials is crucial to the success of the region’s response to mpox.

A persistent challenge across the region is the logistics and supply chain systems necessary to support outbreak response efforts. Several countries, including Cabo Verde and Guinea-Bissau, lack emergency supply mechanisms, cold chain capacity, and transport systems for medical supplies. This results in delayed mobilization of essential resources like PPE, diagnostics, and vaccines, which undermines the overall effectiveness of response efforts. Pre-positioning supplies in key strategic locations, improving transport infrastructure, and establishing functional cold chain systems are critical to ensuring that all member states are adequately equipped to respond to mpox outbreaks. Additionally, enhancing regional stockpile systems and fostering cross-border collaboration on logistics can optimize resource distribution across ECOWAS.

The gaps identified across ECOWAS member states underscore the need for sustained investments and interventions to strengthen preparedness and response to mpox and future public health threats. Strengthening surveillance systems, scaling up laboratory capacity, improving IPC measures, and addressing logistics and supply chain issues are essential to improving the region’s overall readiness. A coordinated effort that leverages national and regional resources, builds stronger health systems, and fosters international collaboration will be crucial in ensuring that ECOWAS is better prepared for future outbreaks.

**Recommendations:** to enhance preparedness and management of the mpox outbreak in the ECOWAS region, the West African Health Organization (WAHO) can prioritize the following key activities:

***Develop national preparedness and response plans:*** work with member states to create tailored national plans for mpox that outline clear roles, responsibilities, and protocols for prevention, detection, and response.

***Accelerate training of health workers:*** focus on improving training for healthcare professionals, especially in countries lagging, to enhance mpox surveillance, early detection, and case management.

***Strengthen linkages between rapid response teams (RRTs) and emergency operations centres (EOCs):*** facilitate better coordination and communication between RRTs and EOCs to ensure a unified and efficient response to mpox cases.

***Integrate the private healthcare sector into national surveillance systems:*** encourage member states to include private healthcare providers in national surveillance frameworks to ensure comprehensive data collection and rapid case identification.

***Enhance public engagement and risk communication:*** implement strategies to adapt and translate mpox-related communication materials into local languages, ensuring public awareness and understanding of the disease and preventive measures.

**Limitation:** variability in the quality and reliability of data collected from different countries may affect the accuracy of the assessment. Some countries may have incomplete or outdated information, leading to potential misinterpretations of their readiness levels. The reliance on self-reported data from member states can introduce bias, as countries may overstate their preparedness levels to present a more favorable image. Variations in resources, infrastructure, and healthcare systems across countries can lead to disparities in the findings. The study might not capture the unique challenges faced by specific nations.

## Conclusion

The findings reveal a mixed picture of mpox preparedness across ECOWAS member states. While significant progress has been made in establishing the foundational structures for epidemic and pandemic response, such as risk communication plans, surveillance systems, and coordination mechanisms critical gaps remain that hinder the region’s overall readiness to combat mpox effectively. These gaps range from limited laboratory capacity and inadequate infection prevention measures to insufficient case management protocols and logistics challenges. Infection control, particularly in healthcare settings, and timely case management stand out as areas of immediate concern. The lack of trained healthcare personnel, coupled with inadequate ICU capacity and absent referral facilities, poses significant risks in the face of a large-scale outbreak. Furthermore, while most countries have established risk communication frameworks, the failure to adapt and translate IEC materials limits the reach of public health campaigns, particularly in multilingual contexts. Additionally, the logistics and supply chain issues underscore the fragility of the region’s emergency preparedness infrastructure. The inability to mobilize and distribute essential supplies rapidly due to a lack of focal points, emergency supply mechanisms, and cold chain storage points to critical vulnerabilities in the region’s capacity to handle surges in mpox cases.
